# Supplementary material for: Delivering Two Tumour Antigens Survivin and Mucin-1 on Virus-Like Particles Enhances Anti-Tumour Immune Responses
Source: Vaccines (Basel). 2021 May 6;9(5):463. doi: 10.3390/vaccines9050463 (PMC8148150; doi:10.3390/vaccines9050463)
Supplement: Supplementary file 1 [file vaccines-09-00463-s001.zip › vaccines-1181693-supplementary.pdf]

Supplementary Materials

Protein View: 17079_Seq1

(Seq1_17079) RHDV VP60

**Database:**	UserDB
**Score:**	67410
**Nominal mass (M_r):**	61179
**Calculated pI:**	5.05

Sequence similarity is available as an NCBI BLAST search of 17079_Seq1 against nr.

Search parameters

**MS data file:**	17083_A_B1_71.mgf
**Enzyme:**	Trypsin: cuts C-term side of KR unless next residue is P.
**Variable modifications:**	Carbamidomethyl (C), Deamidated (NQ), Oxidation (M)

Protein sequence coverage: 12%

Matched peptides shown in ***bold red***.

1 **MTVSEFLKLG GSEGKAR**AAP QGEAAGTATT ASVPGTTTDG MDPGVVATTS
51 VITAENSSAS IATAGIGGPP QQVDQQETWR TNFYYNDVFT WSVADAPGSI
101 LYTVQHSPQN NPFTAVLSQM YAGWAGGMQF R**FIVAGSGVF GGR**LVAAVIP
151 PGIEIGPGLE VR**QFPHVVID AR**SLEPVTIT MPDLRPNMYH PTGDPGLVPT
201 LVLSVYNNLI NPFGGSTSAI QVTVETRPSE DFEFVMIRAP SSKTVDSISP
251 AGLLTTPVLT GVGNDNRWNG QIVGLQPVPG GFSTCNRHWN LNGSTYGWSS
301 PR**FADIDHR**K GSASYTGSNA TNVLQFWYAN AGSAIDNPIS QVAPDGFPDM
351 SFVPFNGPGI PAAGWVGFGA IWNSNSGAPN VTTVQAYELG FATGAPGNLQ
401 PTTNTSGAQA VAKSIYAVVT GTAQNPAGLF VMASGIISTP NASAITYTPQ
451 PDR**IVTTPGT PAAAPVGKNT PIMFASVVR**R TGDVNATAGS VNGTQYGTGS
501 QPLPVTIGLS LNNYSSALMP GQFFVWQLTF ASGFMEIGLS VDGYFYAGTG
551 ASTTLIDLTG LIDVRPVGPR PSKSTLVFNL GGTANGFSYV

Figure S1. Mass spectrometry results for surv.VLP.

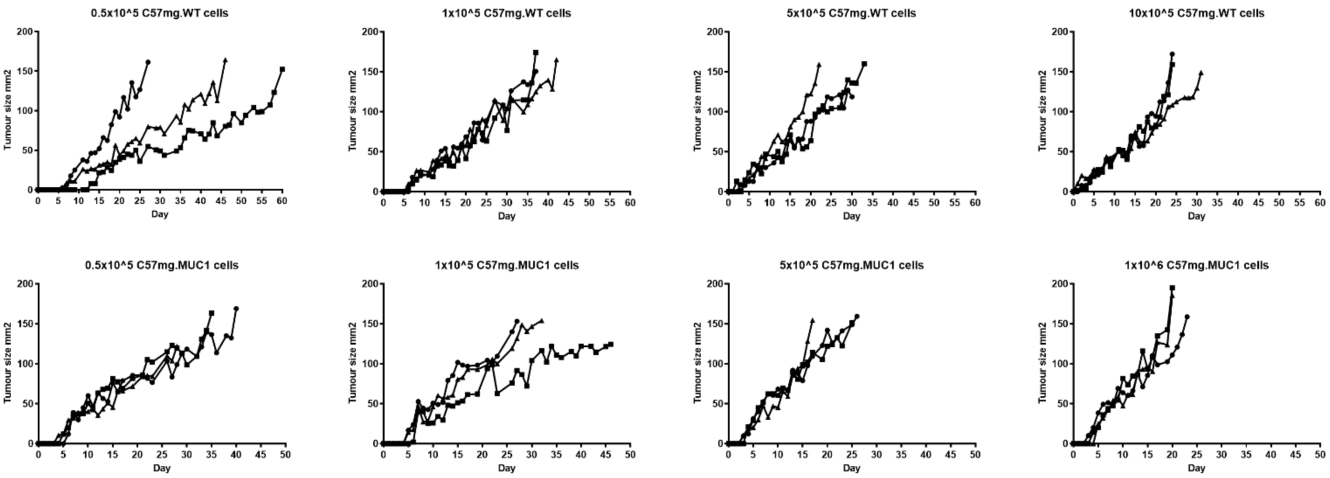

Figure S2. Titration of C57mg.WT and C57mg.MUC1 cells. To establish the optimal number of tumour cells required for tumour inoculation, increasing numbers of C57mg.WT cells (top row) or C57mg.MUC1 cells (bottom row) were implanted into the mammary fat pad of C57/BL6 mice (n = 3 per group). Tumour size was routinely monitored to determine tumour growth kinetics and the optimal cell number for tumour inoculation. Inoculums of 0.5x10^5 C75mg.MUC1 and 1x10^5 C57mg.WT cells were selected for use in therapeutic tumour trials.

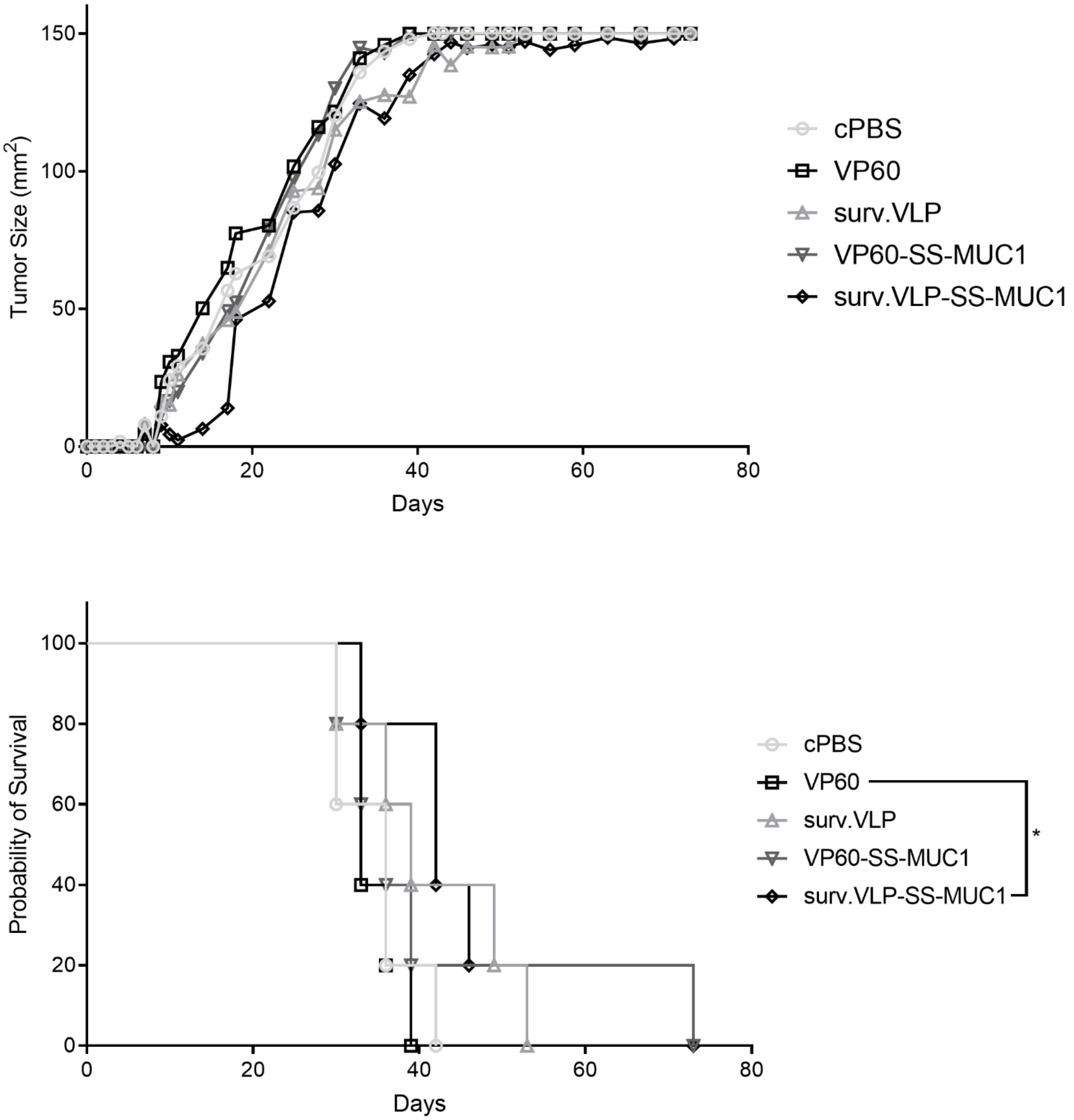

Figure S3. Therapeutic tumour trial of C57mg.WT cells in C57BL/6 mice. C57/BL6 mice with n = 10 mice per treatment group were injected with C57mg.WT tumour cells in the mammary fat pad. Once tumours were palpable, mice were vaccinated with VLP delivering one or two antigens (surv.VLP, VP60-SS-MUC1, or surv.VLP-SS-MUC1), empty VLP controls (VP60) or given sham treatment (cPBS). (a) Tumour growth and (b) survival of mice per treatment group. Statistical significance was determined by a Log-rank (Mantel-Cox) test. *$p < 0.05$.
